# In vivo inhibition of epileptiform afterdischarges in rat hippocampus by light‐activated chloride channel, stGtACR2

**DOI:** 10.1111/cns.14029

**Published:** 2022-12-08

**Authors:** Anirudh R. Acharya, Lars Emil Larsen, Jean Delbeke, Wytse J. Wadman, Kristl Vonck, Alfred Meurs, Paul Boon, Robrecht Raedt

**Affiliations:** ^1^ 4BRAIN Team, Department of Head and Skin, Faculty of Medicine and Health Sciences Ghent University Ghent Belgium

**Keywords:** afterdischarges, chloride, GtACR2, hippocampus, optogenetics, seizures

## Abstract

**Aims:**

The blue light‐sensitive chloride‐conducting opsin, stGtACR2, provides potent optogenetic silencing of neurons. The present study investigated whether activation of stGtACR2 in granule cells of the dentate gyrus (DG) inhibits epileptic afterdischarges in a rat model.

**Methods:**

Rats were bilaterally injected with 0.9 μl of AAV2/7‐CaMKIIα‐stGtACR2‐fusionred in the DG. Three weeks later, afterdischarges were recorded from the DG by placing an optrode at the injection site and a stimulation electrode in the perforant path (PP). Afterdischarges were evoked every 10 min by unilateral electrical stimulation of the PP (20 Hz, 10 s). During every other afterdischarge, the DG was illuminated for 5 or 30 s, first ipsilaterally and then bilaterally to the PP stimulation. The line length metric of the afterdischarges was compared between illumination conditions.

**Results:**

Ipsilateral stGtACR2 activation during afterdischarges decreased the local field potential line length only during illumination and specifically at the illuminated site but did not reduce afterdischarge duration. Bilateral illumination did not terminate the afterdischarges.

**Conclusion:**

Optogenetic inhibition of excitatory neurons using the blue‐light sensitive chloride channel stGtACR2 reduced the amplitude of electrically induced afterdischarges in the DG at the site of illumination, but this local inhibitory effect was insufficient to reduce the duration of the afterdischarge.

## Graphical abstract

Electrical stimulation of the perforant path in the rat model causes afterdischarges to appear bilaterally (yellow arrows) in hippocampal‐parahippocampal structures (black box) (EC—Entorhinal cortex; DG—Dentate Gyrus; CA1‐CA3—Hippocampus proper). Activation of optogenetic chloride channel (stGtACR2) in DG (blue box) was expected to reduce afterdischarge propagation (blue arrow and gray box). Activation of stGtACR2 was observed to reduce amplitude of afterdischarge activity only at the illuminated site (blue box) without affecting afterdischarge occurring at contralateral hemisphere

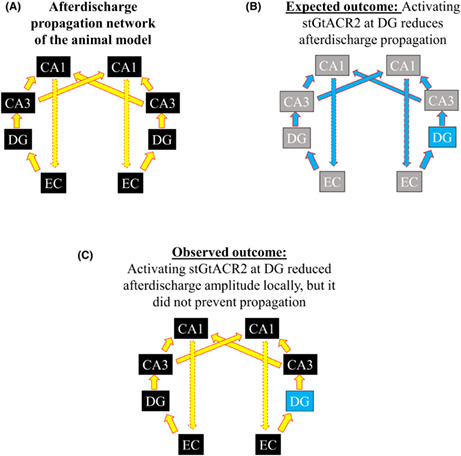

## 1 INTRODUCTION

Seizures are hypothesized to occur due to an excitation‐inhibition imbalance,^1^, ^2^ which emerges due to various etiological conditions.^3^ Hence, the most common approach to prevent seizures is to reduce the overall excitability of the network by treating patients with antiepileptic drugs (AEDs), which act via different mechanisms, such as modulating voltage‐gated ion channels, potentiating GABA_A_‐mediated neuronal inhibition, or acting on various other targets that either directly or indirectly affect neurotransmission.^4^, ^5^ However, treatment with AEDs can cause side effects, and in approximately 30% of epileptic patients, AEDs are ineffective. These patients suffer from medication‐resistant epilepsy.^6^ Therefore, alternative treatment strategies are needed. When resective surgery is not a therapeutic option (e.g., seizures originating from the eloquent cortex^7^) neurostimulation strategies, such as deep brain stimulation^8^, ^9^, ^10^ and vagus nerve stimulation^11^, ^12^ are considered. Additionally, new developments such as responsive neurostimulation (RNS) (i.e., stimulation delivery upon detection of seizure onset) have shown promising outcomes.^13^, ^14^, ^15^ The RNS approach is believed to cause minimal perturbation of normal brain function while curtailing the seizures. However, electrical stimulation nonspecifically modulates neurons near the stimulation contact and can result in side effects (e.g., fatigue and depression^16^). Optogenetics is a technique that is frequently used in fundamental neuroscience research and involves inducing the expression of light‐sensitive proteins (opsins) in neurons of interest to modulate their activity on demand by the administration of light. Several features of optogenetics make it a powerful responsive intervention for seizure suppression. First, this technique allows specific modulation of only the neurons of interest based on the choice of the gene promoter driving opsin expression. For example, the CaMKIIα promoter is expressed only in excitatory neurons,^17^, ^18^ and because of its short base pair length, it can be integrated with a selected transgene in the viral vector to transduce neurons in vivo.^19^ Second, opsins can be used to either activate or inhibit the neurons of interest with a high degree of spatiotemporal precision provided by light delivery. GtACR2 is a highly photosensitive (EPD50: 0.05 mW/mm^2^) and selective light‐activated anion channel that can inhibit mature neurons by shunting the membrane potential towards equilibrium potential of chloride.^20^, ^21^ Soma‐targeted GtACR2 (stGtACR2) is a genetically engineered variant of GtACR2 with a K_v_2.1 motif at the C‐terminal of the GtACR2 peptide. This adaptation results in increased cell surface expression at the proximal dendrites and soma while reducing its expression in axons by approximately 50%. This is important because chloride concentration can be locally increased in axons, so that activation of axonal chloride channels can result in chloride efflux, leading to paradoxical depolarization and neuronal excitation.^21^, ^22^

Optogenetics has been successfully implemented in various animal models of neurological disorders including epilepsy.^23^, ^24^ Light‐controlled, on‐demand neuronal inhibition is feasible, resulting in seizure suppression with minimum latency. Previously, chloride‐conducting inhibitory pumps such as NpHR and Jaws have been used to inhibit neurons in both in vitro and in vivo seizure models.^25^, ^26^, ^27^, ^28^, ^29^, ^30^, ^31^ In the present study, stGtACR2 was used to induce focal inhibition of dentate gyrus (DG) neurons in an in vivo rat model of electrically evoked hippocampal afterdischarges (repetitive, high‐amplitude spiking activity on hippocampal local field potentials [LFP]). The DG is a crucial structure in the temporal lobe and is involved in various functions, such as encoding, pattern separation, and memory retrieval.^32^ Normally, granule cells, the principal excitatory neurons of the DG, respond sparsely to afferent inputs from the entorhinal cortex. This feature enables the DG to act as a filter for signals entering the hippocampal circuitry; hence, it is referred to as the “Dentate Gate”.^33^, ^34^ Various pathological modifications of the DG, such as mesial temporal sclerosis, hilar cell loss, mossy fiber sprouting, granule cell dispersion, and other molecular modifications, are believed to jeopardize the filtering function of the DG and make the hippocampal circuitry hyperexcitable.^34^ Therefore, the restoration of granule cell inhibition in the DG may prevent seizures.^33^ In this study, we attempted to inhibit excitatory neurons in the DG using stGtACR2 and evaluated the effects of focal inhibition on electrically evoked afterdischarges. Our study provides insights into the applicability of the optogenetic chloride channel stGtACR2 for seizure control.

## 2 MATERIALS AND METHODS

### 2.1 Animals

Adult male Sprague–Dawley rats (8 weeks old, 250–400 g, *n* = 12) (Envigo) were used. The animals were housed in a temperature‐ and humidity‐regulated room with a 12 h light/dark cycle (lights “on” between 8 a.m. and 8 p.m.). Water and food were provided ad libitum. All procedures were approved by the Animal Experimental Ethical Committee of Ghent University (ECD 19/38). The treatment and care complied with the ARRIVE guidelines.

### 2.2 Viral vector injection

For injecting the viral vector, rats were anesthetized with isoflurane (5% in medical O_2_ for induction and 2% for maintenance, 1 L/min). The body temperature was maintained at 35 ± 1°C using a heating pad with a rectal probe for temperature feedback. Upon exposure of the skull, the rats were bilaterally injected with 0.9 μl of AAV2/7‐CaMKIIα‐0.4‐intron‐stGtACR2‐fusionred (6.76 × 10^11^ genome copies/ml, Addgene plasmid # 105669) (Leuven Viral Vector Core) using a Hamilton syringe (Model 7001, Hamilton Co., 0.1 μl/min flow rate) in DG (−5.0 mm anteroposterior, ±3.0 mm mediolateral to bregma, −3.0 mm dorsoventral to brain surface). After the injection, the needle was left in place for 5 min to minimize backflow of the viral vector solution due to removal of the injection needle. Postoperatively, meloxicam (1 mg/kg) was administered subcutaneously for analgesia.

### 2.3 Surgical procedures for electrophysiological recordings

All subsequent data were obtained three weeks later during a single recording session for each rat under isoflurane anesthesia (1.5%–2%, 1 L/min) while maintaining a constant body temperature (35 ± 1°C). Upon exposure of the skull, an epidural screw electrode (1.25 mm diameter) was placed in the left frontal bone and used as the ground/reference electrode. Using stereotaxic procedures, the recording optrode was placed in the DG (AP: −5.0 mm, ML: ±3.0 mm, DV: −3.0 to −3.3 mm) and a stimulation electrode targeted the perforant path (PP) (AP = Lambda, ML: ±4.1 mm, DV: −2.0 to −3.0 mm). The optrode was custom‐made by gluing a multimode optical fiber (200 μm diameter, 0.39 NA, Thorlabs) 500 μm proximal to the distal contact of the bipolar recording electrode. The bipolar recording electrode comprised two twisted polyimide‐coated stainless‐steel wires with a diameter of 70 μm and a tip separation of 1 mm (California Fine Wire). A stimulation electrode was also prepared using two twisted polyimide‐coated stainless steel wires with a diameter of 120 μm and a tip separation of 900 μm (California Fine Wire). The dorsoventral position of the optrode was fine‐tuned by using auditory feedback. The depths of the optrode and stimulation electrode were further adjusted using electrophysiological feedback: the optrode position was optimized until the distal contact of the electrode was below the granule cell layer of DG where fEPSP appear as a positive voltage wave superimposed with a negative population spike (PS). Input–output (IO) relationships were determined by applying electrical pulses with increasing current intensity (100 to 1000 μA in steps of 100 μA) and an interval of 10 seconds between stimuli. This procedure was repeated four times. For each rat, the stimulation intensity resulting in 90% of the maximal PS amplitude (I_90_:439 ± 41 μA) was selected to test the effect of stGtACR2 activation on EPs. The afterdischarges were induced by electrically stimulating the PP at 20 Hz for 10 s. The threshold for afterdischarge induction was determined by increasing the current intensity in steps of 50 μA every 3 min. After determining the threshold (229 ± 53 μA), the current intensity was raised by 100 μA, which was used to evoke afterdischarges at the onset of every 10‐min period. At every second period, 5 or 30 s continuous illumination was applied immediately after the cessation of electrical stimulation.

### 2.4 Instrumentation for electrophysiology and optogenetics

Analog signals were sampled at 10 kHz, amplified 100 times, high‐pass filtered at 0.1 Hz, (±0.1 V input range and 1.526 μV/bit input‐referred resolution) using a USB‐6259 NI‐DAQ card (National Instruments). The NI‐DAQ card was connected to a computer that was used to control stimulation and data acquisition using custom‐made software. A TTL‐controlled laser (SLOC Lasers) was used to deliver blue light (473 nm) through the optrode. Prior to implantation, the laser output settings were defined to achieve four increasing light power densities (LPDs) at the optical fiber tip: 64, 127, 191, and 255 mW/mm^2^. This was checked using a handheld light power meter (PM100A + S120VC; ThorLabs). A constant‐current linear stimulus isolator (Digitimer) generated the charge‐balanced square‐wave pulses (200 μs per phase) needed to obtain the EPs and afterdischarges.

### 2.5 Experimental paradigms

In Experiment 1, the effect of stGtACR2 activation on EPs was observed in five rats. The experiment was performed in both hemispheres. First in the right hemisphere, followed by the left hemisphere (ten recordings in total). To determine the minimal LPD required for robust inhibition of neurons, DG EPs were evoked every 10 s at I_90_, and every second EP was paired with a 10 ms light‐pulse terminating on the PS. Four LPDs (64, 127, 191, and 255 mW/mm^2^) were tested for inhibitory effects. The lowest LPD required for maximal inhibition of PS was determined and used in the remaining experiments.

**FIGURE 1:**
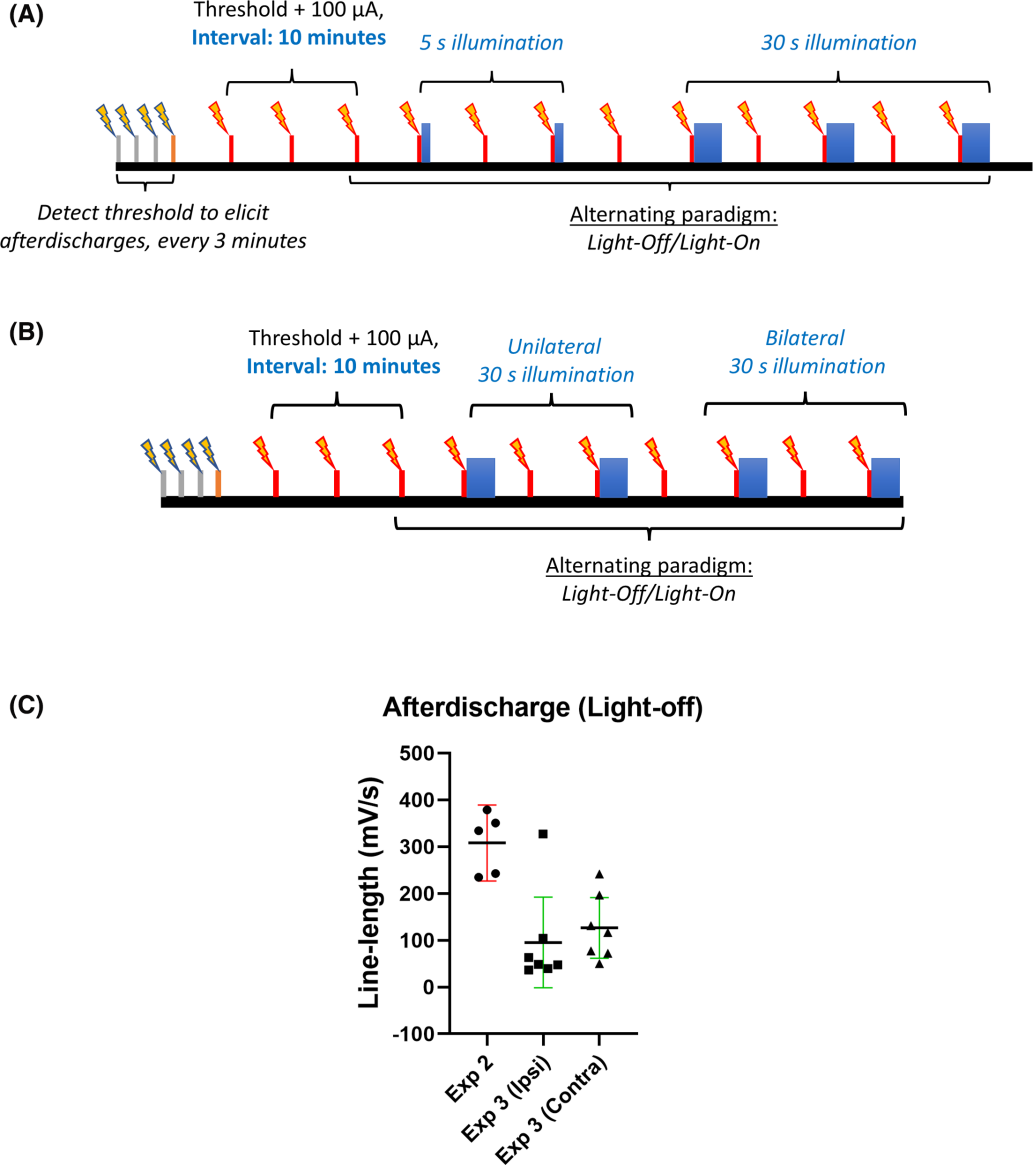
Illustration of experimental paradigms to induce afterdischarges. Thin gray, orange, and red bars with bolts represent 20 Hz, 10 s electrical stimulation. Blue bars represent illumination for 5 and 30 s. (A) Paradigm for unilateral recordings (B) Paradigm for bilateral recordings (C) Afterdischarge line length of individual rats (data points, Mean ± 95% CI) during light‐off conditions for experiment 2 (Exp 2, *n* = 5) and experiment 3 (Exp 3, ipsilateral and contralateral to electrical stimulation site, *n* = 7).

In Experiment 2, afterdischarges were evoked in the same five rats after performing Experiment 1 by PP stimulation in the left hemisphere. The effect of activating stGtACR2 in the ipsilateral DG on afterdischarge activity was evaluated using an optrode implanted above the DG in the left hemisphere (*n* = 5). Three iterations of alternating the light‐off and light‐on paradigms were recorded for each rat (Figure 1A).

In Experiment 3, optrodes were implanted bilaterally above the DG to test the effect of unilateral and bilateral activation of stGtACR2 on afterdischarge activity, both ipsilaterally and contralaterally, at the site of the PP stimulation (*n* = 7). Two iterations of alternating light‐off and light‐on paradigms were recorded for each rat (Figure 1B).

### 2.6 Histology

At the end of the experiments, rats were deeply anesthetized with an overdose of thiobarbital (180 mg/kg i.p.) and transcardially perfused with ice‐cold phosphate‐buffered saline (PBS) followed by paraformaldehyde in 0.1 M phosphate buffer solution (4%, pH 7.4). The brains were isolated and post‐fixed overnight in 4% paraformaldehyde. After cryoprotection in 30% sucrose, the brains were snap‐frozen in 2‐methylbutane, cooled with liquid nitrogen. Coronal sections of 40 μm thickness were collected using a cryo‐microtome (Leica) set at −20°C. The sections were then washed twice with PBS and incubated for 30 and 60 min in 0.5% and 1% H_2_O_2_. Next, they were permeabilized and blocked in 0.2% Triton‐X‐100/0.4% fish skin gelatin (blocking buffer), after which they were transferred to the primary antibody solution (rabbit polyclonal anti‐tRFP, 1:1000, Evrogen AB233) and incubated overnight at 4°C. The next day, the sections were washed twice in blocking buffer, followed by 1 h in a secondary antibody (goat antirabbit Alexa Fluor 594, 1:1000, Abcam ab150088). After rinsing twice with PBS, DAPI (4′,6‐diamidino‐2‐phenylindole) was added for nuclear staining (1 μg/ml, Sigma‐Aldrich). The slices were mounted on glass slides, Vectashield® antifade mounting medium (Vector Laboratories) was applied, and a coverslip was placed. Images were obtained using a fluorescence microscope (AxioCAM HR, Carl Zeiss).

### 2.7 Data analysis

All statistical analyses were performed using jamovi 2.0 modules (The jamovi project, 2021) (www.jamovi.org), and graphs were prepared using Prism software (Graphpad). The mean and individual data points are plotted. The Kolmogorov–Smirnov test was used to check the normal distribution of the data. The EPs were recorded and analyzed using a custom‐made MATLAB program (MathWorks). The PS amplitude was measured by plotting the tangent line from the middle of the negative peak to the middle of the line drawn between the two positive peaks of fEPSP. The relative changes in the PS amplitude due to stGtACR2 activation were assessed by calculating the ratios between the light‐on and light‐off conditions (light on/light off) for each individual animal. To determine whether there was an effect of increasing LPD on the level of PS inhibition, repeated‐measures ANOVA with Tukey's test for post hoc comparison was used.

The start of afterdischarge was defined as the time point immediately after electrical stimulation when the amplitude exceeded twice the baseline EEG amplitude. The end of the afterdischarge was defined as the time point at which the amplitude decreased below twice the baseline EEG amplitude for more than 1 s. The time between the start and end was considered as its afterdischarge duration.^35^ The line length per second of the LFP, measured at the DG, was calculated during the last 20 s before and the first 30 seconds after stimulation of the PP for induction of afterdischarges. Different illumination conditions were evaluated for their effect on afterdischarges by calculating the relative changes in the line length of the local field potential post‐ versus prestimulation.

For unilateral recordings, the relative changes in LFP line lengths under light‐on and light‐off conditions were compared using a paired *t*‐test. For simultaneous bilateral recordings, the relative changes in line lengths in the ipsilateral and contralateral hemispheres under different illumination conditions were compared using repeated‐measures two‐way ANOVA with Tukey's test for post hoc comparison. Differences were considered statistically significant at *p* < 0.05.

## 3 RESULTS

### 3.1 Experiment 1: effect of the applied LPDs on PS amplitude of stGtACR2 expressing rats

**FIGURE 2:**
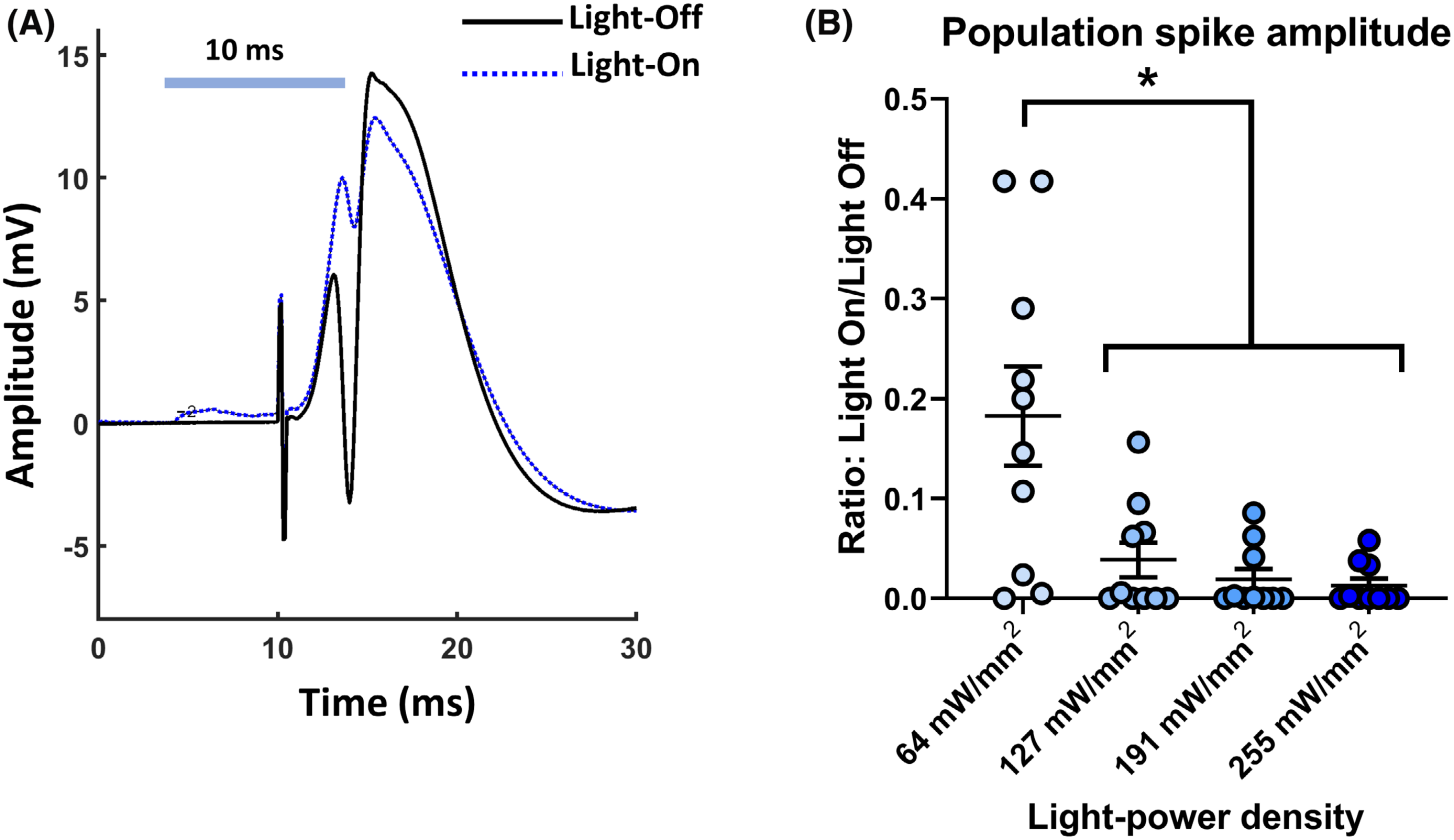
(A) Representative traces of evoked potentials recorded at dentate gyrus in stGtACR2 expressing rat, with and without illumination (127 mW/mm^2^, 10 ms) (B) Increasing light power densities resulted in near complete suppression of population spike (PS) in stGtACR2 expressing rats (*n* = 5, bilateral recordings). *p < 0.05

An LPD‐dependent increase in the inhibitory effect of stGtACR2 activation on the PS amplitude of DG EPs was observed (*F* = 12.60, *p* < 0.005). The magnitude of PS inhibition was significantly lower at 64 mW/mm^2^ than at 127, 191, or 255 mW/mm^2^ (*p* < 0.05). The inhibitory effect on the PS amplitude did not significantly differ between the higher LPDs (Figure 2A,B). Based on these results, we chose to use an LPD of 127 mW/mm^2^ for subsequent experiments.

### 3.2 Experiment 2: effect of illumination on the line length of afterdischarges

**FIGURE 3:**
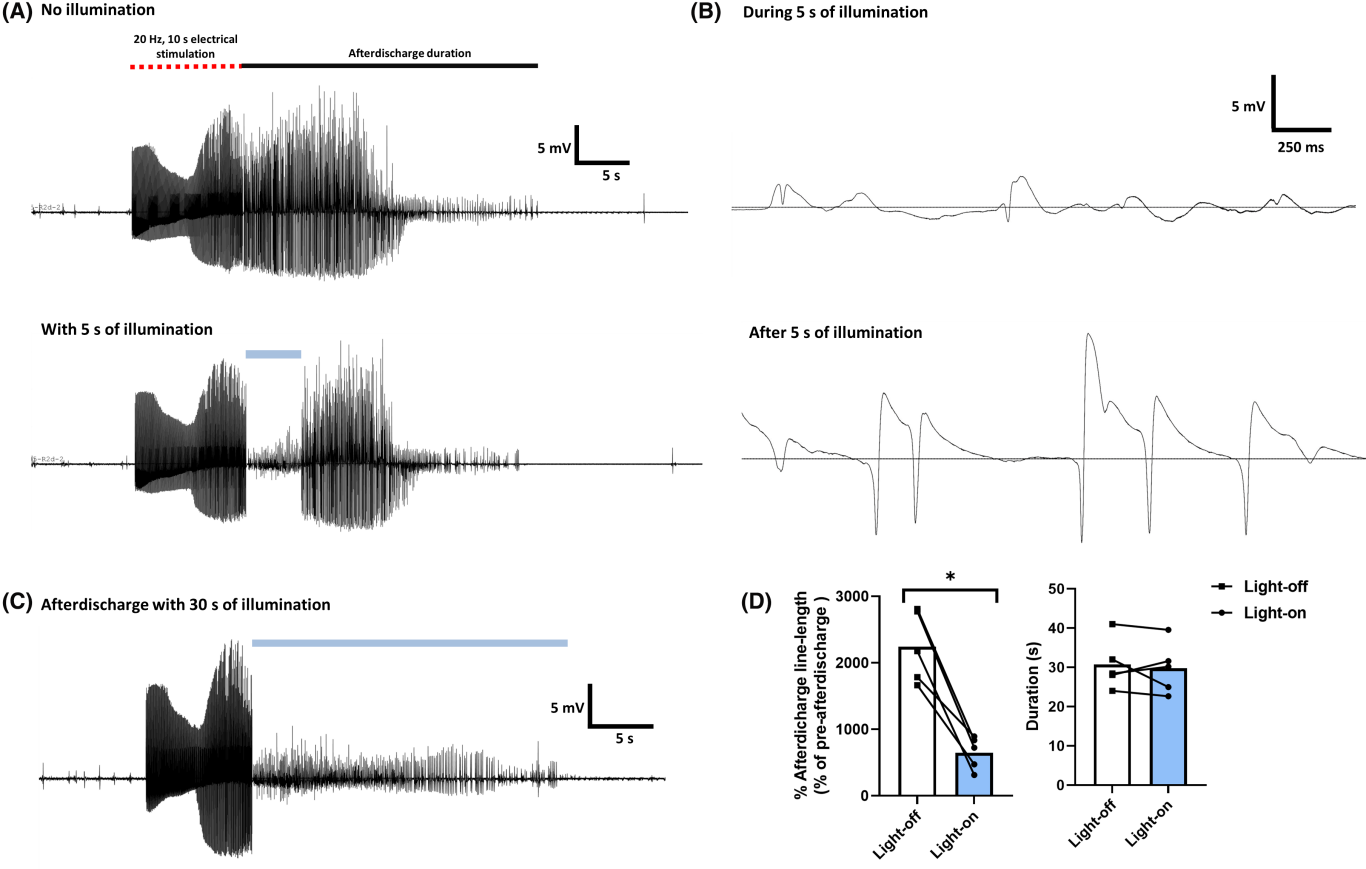
(A) Amplitude of afterdischarges decreased only during 5 s of illumination (B) Same afterdischarge represented in a shorter time scale, during 5 s illumination the polyspike was inhibited, that resumed immediately after (C) Light delivery for 30 s decreased the amplitude of afterdischarges for the duration of illumination, but it did not terminate them (D) Illumination for 30 s significantly decreased the line length metric, but it did not reduce the duration of afterdischarges (*n* = 5, unilateral recordings). *p < 0.05

Activation of stGtACR2 during afterdischarges decreased the local field potential amplitude only during the 5 s illumination period and resumed its polyspike activity immediately thereafter **(**Figure 3A,B **)**. The inhibition of afterdischarge activity was sustained in the case of 30 s of illumination (Figure 3C), with a significant decrease in line length (*t* = 6.85, *p* < 0.005) (Figure 3D). However, it did not terminate or reduce the duration of the afterdischarges.

### 3.3 Experiment 3: effect of illumination on the line length of afterdischarges in the contralateral hemisphere

**FIGURE 4:**
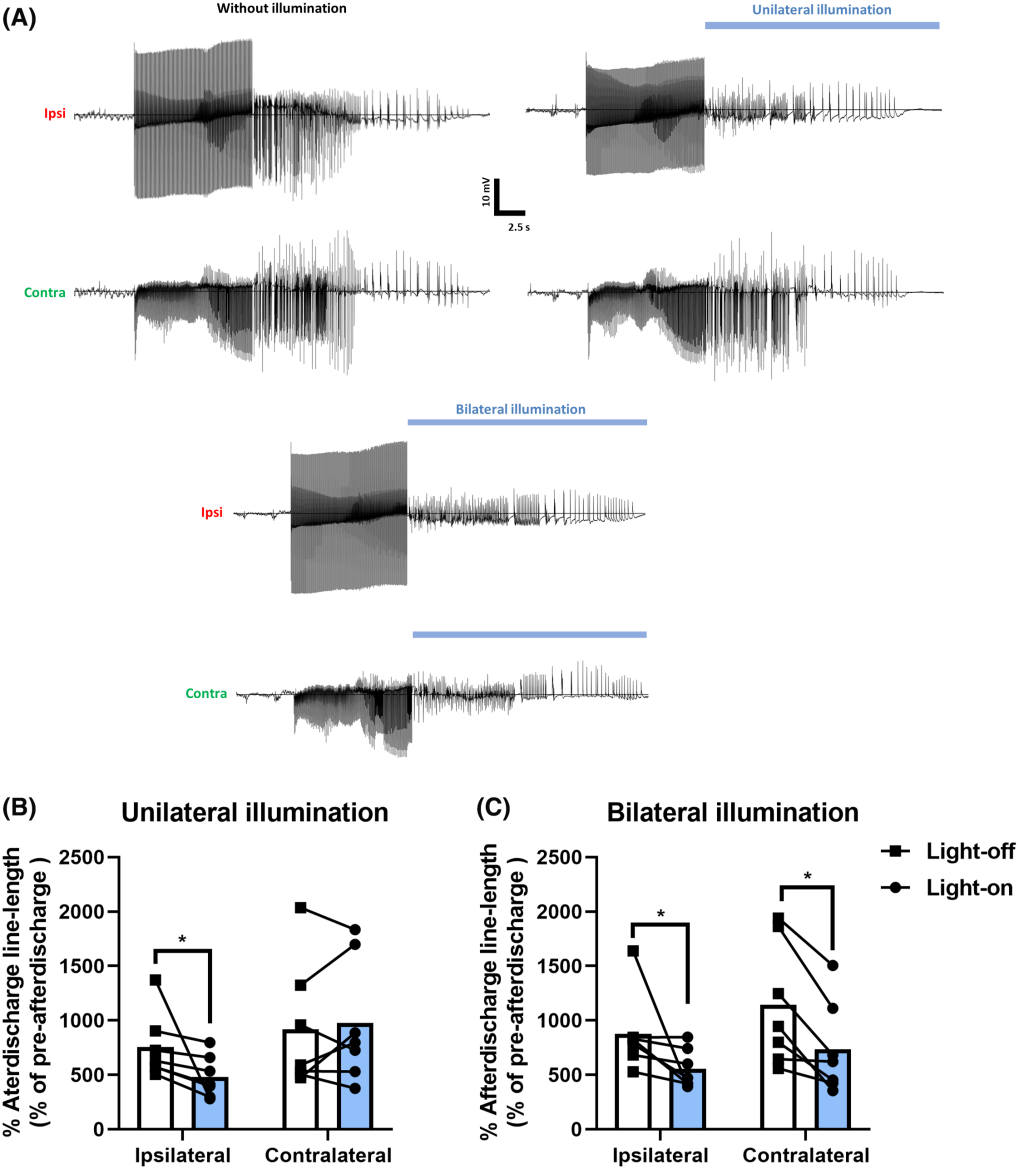
(A) Unilateral illumination decreased the amplitude of afterdischarges ipsilateral to site of illumination and did not affect the afterdischarges occurring at contralateral hemisphere. Bilateral activation of stGtACR2 did not terminate or reduce the duration of afterdischarges. Effect of (B) unilateral (C) bilateral 30 s illumination on afterdischarge line length (*n* = 7, simultaneous bilateral recordings). *p < 0.05

Unilateral electrical stimulation of the PP resulted in the appearance of afterdischarges in both the ipsilateral and contralateral DG **(**Figure 4A **)**. For unilateral illumination, an interaction between the illumination conditions and illumination site was found (*F* = 6.94, *p* < 0.05). The line length significantly decreased only ipsilaterally to the site of electrical stimulation, where illumination was performed (Figure 4B). When illuminated bilaterally, the illumination condition had a significant effect (*F* = 11.23, *p* < 0.05). Bilateral illumination decreased the line length of afterdischarges in both hemispheres (Figure 4C); however, it did not reduce afterdischarge duration.

### 3.4 Histological verification of stGtACR2 expression

**FIGURE 5:**
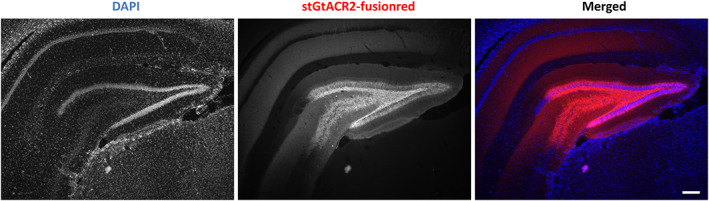
Representative histological image of stGtACR2 expression in DG of rat brain. stGtACR2‐fusionred appearing in red and nuclear‐stain DAPI appearing in blue color, respectively. Scale bar measures 200 μm.

Epi‐fluorescence microscopy three weeks after viral vector injection showed expression of fusionred in the DG granule cell layer and hilus, as well as in the CA3 pyramidal cell layer. The main cell types expressing the CamKIIα promoter in these regions were granule cells, mossy cells, and pyramidal neurons. We found expression in both cell bodies and neurites, such as mossy fibers (Figure 5). No discernible variability in expression quality was observed among the rats.

## 4 DISCUSSION

This study showed that pulses of blue light administered to stGtACR2‐expressing excitatory neurons of the DG reliably decreased the PS amplitude of DG‐evoked potentials induced by electrical stimulation of the PP. Illumination with an LPD of 127 mW/mm^2^ was sufficient to achieve maximal inhibition of DG PS. This LPD not only results in successful inhibition of DG granule cells but also negligible tissue heating,^36^ and we used it to test the effect of this optogenetic inhibition of DG granule cells on epileptiform afterdischarges induced by PP stimulation. Although we found a decrease in seizure line length during illumination, we were unable to terminate or shorten seizures. This is in contrast to previous studies that have shown successful seizure termination in response to optogenetic inhibition or excitation of excitatory or inhibitory neurons in various preclinical models of seizures.^26^, ^28^, ^33^, ^37^, ^38^, ^39^, ^40^, ^41^ The contrasting observations in our study compared to those in previous studies might be related to differences in the experimental approach. Afterdischarges induced by PP stimulation are not focal, but simultaneously recruit a large bilateral temporal lobe network.^42^, ^43^, ^44^ Apart from the DG, afterdischarge activity has been observed in the hippocampus proper, subiculum, and entorhinal cortex.^42^, ^43^, ^44^ Our findings indicate that activation of stGtACR2 in the DG subfield had a focal inhibitory effect on afterdischarges, but did not prevent their propagation. Thus, optogenetic inhibition of relatively small volumes of neurons in the DG is unlikely to suppress afterdischarge beyond the illuminated site. This is believed to be due to the limited connectivity of DG granule cells in the dorsoventral axis of the hippocampus and a lack of projections to the contralateral hemisphere.^33^, ^45^

Seizure suppression achieved in various preclinical models is likely to be dependent on the targeted cell type. CA3 pyramidal neurons have extensive axonal projections (Schaffer collaterals, SC) along the longitudinal axis of the hippocampus, including the contralateral hemisphere.^45^, ^46^ Interventions targeting these regions have demonstrated the successful interruption of electrically induced afterdischarges. For example, optogenetic inhibition of principal excitatory CA3 neurons using Jaws in the PP kindling model significantly reduced afterdischarge duration and behavioral severity.^31^ Additionally, in an anesthetized rat model of afterdischarge (SC stimulation), high‐frequency electrical stimulation of the SC prevented afterdischarge propagation to the CA1 subfield by desynchronizing neurons from epileptiform activity.^47^ Several studies have indicated the potent seizure‐suppressing effects of activating GABAergic interneurons (i.e., indirectly inhibiting excitatory neurons) in various preclinical models. However, depending on the interneuronal axonal projections, such an optogenetic inhibitory approach can affect larger volumes of the brain beyond the illuminated site.^48^, ^49^ For example, unilateral optogenetic activation of interneurons has been observed to suppress seizures bilaterally by GABA_A_R activation in different seizure models, such as IHKA and 4‐AP,^26^, ^38^, ^50^ although these interneurons constitute less than 5% of hippocampal neurons.^26^ These data suggested that neurons with extensive connections and projections can suppress seizures beyond the illuminated site. However, we cannot exclude the possibility that inhibition of other cell types, such as DG mossy cells (which might express stGtACR2 because of CaMKIIα promoter‐driven expression), contributed to the failure of complete afterdischarge suppression in our experiments. Another limitation of our study is the lack of unbiased stereological cell count data and a computational model of light distribution to estimate the number of inhibited stGtACR2‐expressing neurons. Plausibly, stGtACR2‐mediated optogenetic inhibition might have resulted in afterdischarge termination if a larger DG volume and/or number of granule cells had been targeted. To achieve this, an approach involving multiple illumination sites targeting the seizure network can be attempted using this rat model.

A limitation of our study design was that we tested the afterdischarge‐suppressing effect of stGtACR2 only in male rats under anesthesia. Sex‐specific differences in brain metabolism, vasculature, gene expression,^51^, ^52^, ^53^, ^54^, ^55^, ^56^ and seizure susceptibility^57^ have been observed. In addition, isoflurane anesthesia alters the excitability and membrane potential of several neuronal types,^58^, ^59^ which may influence experimental outcomes. To make reliable conclusions about the potential of stGtACR2 as a tool to suppress seizures, future experiments need to be done in chronic epilepsy models in freely‐moving conditions in both male and female rats. In epileptic conditions, several findings indicate changes in internal chloride concentrations (in both preclinical^60^, ^61^, ^62^, ^63^ and clinical^62^, ^64^, ^65^, ^66^, ^67^ studies), rendering GABA_A_R‐mediated inhibition less effective.^68^, ^69^, ^70^ Such changes can occur because of alterations in the mRNA expression of chloride transporters, such as NKCC1 (upregulation) and KCC2 (downregulation), depending on the brain region^65^ and/or time of intervention.^63^ An increase in internal neuronal chloride concentration causes a positive shift in the chloride equilibrium potential, weakening chloride‐mediated inhibition. Therefore, stGtACR2 may be less effective at inhibiting neurons in epileptic conditions. One way to further optimize the use of GtACR opsins in epileptic conditions is to use them in combination with proton pumps, such as Arch.^71^ This creates a driving force for chloride extrusion from neurons when GtACR and Arch are simultaneously activated. Therefore, it can also be used to reduce internal chloride concentration and restore physiological chloride channel‐mediated inhibition in epileptic conditions.^71^

## AUTHOR CONTRIBUTIONS

AA obtained the data. AA, LL, and RR analyzed the data. AA prepared the manuscript. AA, LL, JD, WW, KV, AM, PB, and RR contributed to the study design and analysis plan. All authors reviewed the manuscript.

## CONFLICT OF INTEREST

The authors declare that the research was conducted in the absence of any commercial or financial relationships that could be construed as a potential conflict of interest.

## Data Availability

The data that support the findings of this study are available from the corresponding author upon reasonable request.
